# Impact of Smoking Habit on Peri-Implant Indicators following Different Therapies: A Systematic Review

**DOI:** 10.3390/bioengineering9100569

**Published:** 2022-10-18

**Authors:** Davide Farronato, Lorenzo Azzi, Luca Giboli, Vittorio Maurino, Gianluca Martino Tartaglia, Marco Farronato

**Affiliations:** 1 Department of Medicine and Surgery, School of Dentistry, University of Insubria, 21100 Varese, Italy; 2 Department of Biomedical, Surgical and Dental Sciences, School of Dentistry, University of Milan, 20100 Milan, Italy; 3 UOC Maxillo-Facial Surgery and Dentistry Fondazione IRCCS Cà Granda, Ospedale Maggiore Policlinico, Department of Orthodontics, School of Dentistry, University of Milan, 20122 Milan, Italy

**Keywords:** smoking, dental implants, peri-implantitis, mucositis

## Abstract

Peri-implant disease and its treatment is becoming a major concern for clinicians as the number of implants placed each year is rising. Smoking is a common habit, and it is associated with an increased risk of developing peri-implant disease. The role of smoking in the response to peri-implant treatment has never been investigated. Searches were conducted in electronic databases to screen articles published until August 2021. The included studies had at least two groups of patients: peri-implant disease only or peri-implant disease and smoking status. Outcomes of interest included plaque index (PI), probing depth (PD), bleeding on probing (BoP), radiographic crestal bone loss (CBL), and analysis of peri-implant sulcular fluid. Seven hundred and forty-nine articles were found in the databases, only 71 articles potentially qualified. A total of seven studies with a minimum follow-up of six months were included. There is no homogeneity in the diagnosis, smoker definition and treatment proposed. All surgical and non-surgical treatment have statistically significantly different outcomes in smokers and nonsmokers. Recognizing this study’s limitations, we conclude that smoking might play a significant role on the outcome of peri-implant disease treatment. None of the proposed treatments appear to be significantly more effective.

## 1. Introduction

Titanium dental implants have been in use for over 50 years and are a highly successful treatment option for the replacement of missing teeth [1,2]. 

The initial stability is crucial for the survival of an implant, several techniques and protocol have been studied to enhance the early stages of healing and osseointegration [3].

Equally, the long-term success of dental implants is strictly dependent on the maintenance of healthy tissues around them [4,5]. The late complications most often associated with dental implants are inflammatory conditions of the soft tissues and of the bone surrounding implants and their prosthetic components [6]. Those conditions need to be defined and differentiated from a state of peri-implant health.

The classification of the peri-implant diseases proposed by Caton et al. [7] differentiate 4 different conditions: 

Peri-implant health

Peri-implant mucositis

Peri-implantitis

Peri-implant soft and hard tissue deficiencies

Peri-implant disease is a collective term for inflammatory reactions in the tissues surrounding an implant. Peri-implant mucositis is used to describe the presence of inflammation in the mucosa at an implant with no signs of loss of supporting bone. Peri-implantitis, in addition to mucosal inflammation, is characterized by irreversible progressive damage of the supportive bone [8]. 

Dental implants perforate the mucosa and are continually exposed to the oral microflora. Oral bacteria colonize dental implant surface, forming a microbial biofilm over its supra and subgingival components. The importance of specific microbiological factors in the development of peri-implant disease is controversial: a complex interplay between the bacterial challenge and host related factors probably determines the development and progression of the disease [9,10,11]. 

While the infectious nature of peri-implantitis is widely accepted [12], other factors and systemic conditions have negative effects on long-term implant survival. Among those smoking [13], lack of prophilaxis [13,14], history of periodontitis [13,14], diabetes mellitus [13,15], are classified on a medium and medium-high risk level. It is agreed among researchers that an effective preventive regime (supportive therapy) is necessary to maintain the health of peri-implant tissues. 

The diagnostic definition of peri-implant health is based on absence of peri-implant signs of soft tissue inflammation such as redness, swelling, suppuration or profuse bleeding on probing (BoP) and absence of additional bone loss following initial healing [16].

In order to early detect peri-implantitis (PI), routinary follow-up should be carried on at least every six months [17]. During the routinary follow-up visits the following clinical parameters should be collected [16]: Plaque index (PI) and calculus, bleeding on probing, probing depth (PD) and Radiological crestal bone loss (CBL). When a pathological condition occurs, some therapeutic interventions should be initiated as soon as possible [18]. 

If any initial stage is detected during the follow-up, the current evidence shows that non-surgical therapies supported by self-performed hygiene techniques are necessary to improve clinical parameters and can successfully treat peri-implant mucositis and maintain a low incidence of developing peri-implantitis [18,19].

When non-surgical treatment fails to eliminate the causative bacteria or when peri-implantitis is diagnosed, surgical treatment is recommended [16,20,21].

For an effective prevention and treatment of peri-implant diseases it is necessary to evaluate both local and systemic factors affecting the incidence and severity of these conditions [18]. 

Some studies underlined that smoking is considered as one of the factors that have negative influence on the development of peri-implant disease [13,16,22,23].

Tobacco smoking is still a spread habit worldwide and the long-term harmful effects on nearly every organ of the body and risks connected with it have been largely investigated [24], recent studies have also reported that other forms of tobacco consumption such as Heat-not-burn tobacco seem to impact the oral cell populations [25]. 

Smokers have a higher rate of tooth loss than nonsmokers and, therefore, the need for dental implants in smoking patients have increased in incidence [26]. Recent meta-analyses have reported a higher risk of dental implant failure in smokers and a significant increase in the marginal bone loss around implants as compared with nonsmokers [27,28,29,30,31,32]. 

Clinical and peri-implant parameters are compromised in cigarette smokers and recent studies demonstrate the link between this susceptibility to peri-implant disease and increased levels of proinflammatory cytokines such as IL-1beta, TNF alpha and MMP-9 [33].

A smoker is defined as a subject that smokes at least one cigarette daily since, at least, one year [34,35].

However, there is still no consensus on the number of cigarettes smoked and the relationship with implant failure, but heavy smokers may exhibit a higher incidence [36]. 

Heavy smokers are those who smoke 14 or more cigarettes a day, while lighter smokers smoke less then 13 cigarettes per day [37]. 

Previous studies have generally demonstrated that smokers have less favorable healing in response to both non-surgical and surgical periodontal therapy [38,39,40,41,42] and they may also respond differently to peri-implant disease therapy. In fact, the negative effects of smoking on wound healing are mainly caused by chemical substances as nicotine, carbon monoxide, hydrocyanic acid, and nitrogen oxide [43], but the exact mechanisms by which tobacco exerts its influence on healing and more generally on the health of periodontal tissues are not completely elucidated and could be influenced by multiple factors [44,45]. 

To determine the best treatment options for peri-implant disease in smokers we investigated the scientific literature available up to June 2021. A research study was conducted in the Cochrane reviews database and in the PubMed/MEDLINE database to find current evidence and guidelines.

We read and analyzed the results looking for evidence based clinical indications, but to the best of the authors’ knowledge there is no review analyzing this aspect.

The role of smoking as a risk factor needs to be considered closely as recent studies are highlighting the implications of smoking cessation on periodontal and peri-implant status and the outcome of periodontal therapy [46,47].

For the expressed reasons we conducted a systematic review to assess variations in common peri-implant indicators in smokers and nonsmokers following different peri-implant therapies as primary outcome. The secondary outcome was to provide a flowchart as a tool for the dentists and the dental hygienists to be used when in presence of smoking patients affected by peri-implant disease to identify the best approach.

## 2. Materials and Methods

The Preferred Reporting Items for Systematic Reviews and Meta-Analyses (PRISMA) [48] was followed in the preparation of the present systematic review.

The focused aim of the study was to investigate on the evidence about the treatment options for peri-implant disease in smokers and to summarize the findings in an operative flowchart for the clinicians.

Based on the PICOS principle the following focused questions were proposed: 

In patients with osseo-integrated dental implants affected by peri-implant mucositis, does the smoke of cigarettes influence the outcome of non-surgical therapy?

In patients with osseo-integrated dental implants affected by peri-implantitis, does the smoke of cigarettes influence the outcome of surgical therapy?

The protocol for this review was published in the PROSPERO registered on 27 August 2021: CRD42021259718.

### 2.1. Studies Inclusion Criteria

This review included prospective studies with at least two groups of patients: patients with peri-implant disease who are also smokers and patients with peri-implant disease only. 

Patient in these studies had to receive peri-implant therapy and have a minimum follow-up time of six months after therapy. Included studies had to report a separate quantitative analysis of results for smokers and nonsmokers.

Studies in English language published from 1970 until August 2021 were included.

### 2.2. Studies Exclusion Criteria

Studies involving patients under 18 years of age, studies including only nonsmokers (individuals who reported to have never consumed any form of tobacco product), ex-smokers patients or including only smokers. Studies that included patients with systemic diseases like diabetes mellitus, acquired immune deficiency syndrome or other pathologic conditions that causes immunodeficiency. Studies including patients who reported to use antibiotics, corticosteroids, steroid and non-steroidal anti-inflammatory drugs, bisphosphonates or treated with chemotherapy or radiation, within the previous 3 month. Studies that included pregnant or lactating patients. Only controlled trials and restrospective cohorts were considered. Case reports or case series were not considered eligible.

### 2.3. Search Strategy

The search was performed in the following electronic bibliographic databases: 

PubMed/MEDLINE, Scopus, Cochrane Library and ClinicalTrials.gov for studies published until August 2021 (Table 1).

### 2.4. Data Collection

The articles retrieved were screened independently by two of the reviewers, first by analyzing the title and the abstract, in order to identify the studies that could be potentially included according to the selection criteria. Then, the full texts of the potentially eligible studies were examined independently by the reviewers. In case of disagreements the discrepancies were resolved through discussion with a third reviewer.

Data were extracted independently by two review authors and discrepancies were investigated and resolved with the intervention of a third review author, following a calibration procedure performed on the first 20 articles retrieved. 

Data extracted were divided as follows: First author, Journal’s name, Year of publication, Journal quartile, type of treatment, Target sample size/Control sample size, Study arms, reference standard, measured effect, Follow-up and Risk of bias.

### 2.5. Data Synthesis

First, each selected study’s full text was investigated and synthesized alone. Then, in case the studies were sufficiently homogenous between each other, a quantitative synthesis of the findings was carried out. The synthesis is structured around the correlation between smoking and the previously enumerated clinical parameters of peri-implant disease. We provided a description of the interventions, the effects they produced and the differences of the effects in the groups of smokers and nonsmokers. 

The outcome evaluated are the differences at baseline and at follow-up time after treatment of the following variables: implant probing depth (PD), plaque index (PI), bleeding on probing (BoP), radiographic crestal bone loss (CBL), gingival bleeding index and the analysis of peri-implant sulcular fluid. 

### 2.6. Risk of Bias Assessment

In order to assess the risk of bias of the included studies The Newcastle-Ottawa Scale (NOS) for cohort studies was used [49]. Data extrapolated from the selected papers were divided into three sections (domains): selection (1), comparability (2) and outcome (3). Each section was filled on the base of a subset of items according to the official coding manual’s questions. 

Selection domain includes representativeness of the exposed cohort (1a), selection of the non-exposed cohort (1b), ascertainment of exposure (1c) and demonstration that the outcome of interest was not present at start of the study (1d).

Comparability domain evaluates the comparability of cohorts based on the design or analysis (2a).

Outcome domain includes assessment of outcome (3a), adequacy of follow-up time (3b) and adequacy of follow-up cohorts (3c).

A maximum of one star was assigned for each item within the selection and outcome categories, while a maximum of two stars were assigned for comparability.

A lower score stands for a higher risk of bias in the study:

A score of 1 or 2 stars is equivalent to a very high risk of bias, a score of 3, 4 or 5 stars is equivalent to a high risk of bias, a score of 6 stars is equivalent to a medium to high risk of bias, a score of 7 stars is equivalent to a medium risk of bias, a score of 8 stars is equivalent to a medium to low risk of bias, a score of 9 stars is equivalent to a low risk of bias.

## 3. Results

### 3.1. Study Selection

**Figure 1:**
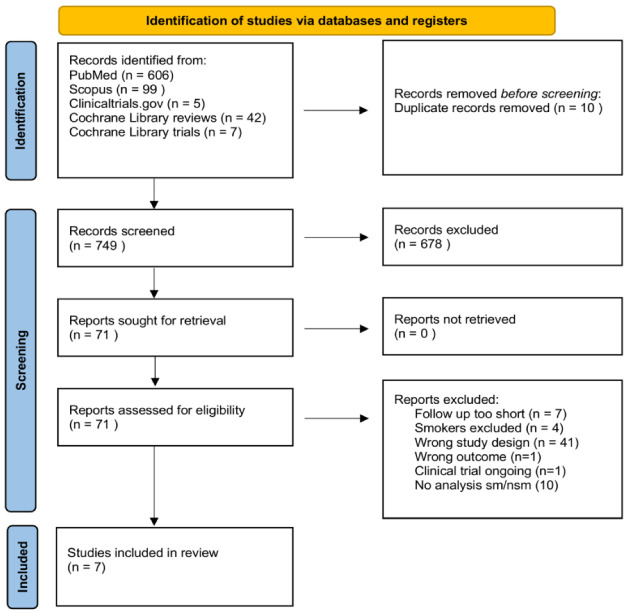
PRISMA flow diagram.

Seven hundred and forty-nine articles were found in the databases (Figure 1). After checking the titles and abstracts, only 71 articles potentially qualified. Seven articles were excluded because the follow-up was too short (less than six months). Four studies were excluded because smokers were not enrolled. Forty-one articles were excluded because of a wrong study design. One article was excluded because the main outcome was not of interest for this research. One study was excluded because it is an ongoing trial, and no provisional results were available from the authors. Ten articles were excluded because they did not provide a separate quantitative analysis for smokers and nonsmokers.

Seven studies were finally eligible for the final selection [50,51,52,53,54,55,56].

### 3.2. Description of Included Studies 

Seven studies were included, published from 2015 to 2020, the follow-up time ranged from 6 months to 54 months (Table 2).

### 3.3. Case Definitions

The diagnostic threshold for peri-implant mucositis varies: Alqahtani B defines peri-implant mucositis as BoP and PD ≥ 3 mm at ≥30% of the sites and CBL up to 2mm. Fernandes-Costa defines peri-implant mucositis as PD up to 5 mm, BoP and no radiographic evidence of bone loss beyond the first two threads of the implant.

The diagnostic threshold for peri-implantitis varies:

Alqahtani A defines peri-implantitis as peri-implant PD of ≥4 mm, and CBL (mesial and/or distal) of ≥3 mm (Moderate smokers >11 day).

Javed defines peri-implantitis as peri-implant BoP in at least 30% sites and PD of at least 4 mm. Al Deeb defines peri-implantitis bleeding and/or suppuration on probing, PD ≥ 6 mm, loss of bone ≥ 3 mm apical of the most coronal portion of the intraosseous part of the implant. Machtei defines peri-implantitis as PD of 5–8 mm, bleeding and/or suppuration on probing and that display radiographic evidence of bone loss of at least 3 mm from the implant shoulder but with at least 2 mm residual bone support. C.M. de Waal defines peri-implantitis as bleeding and/or suppuration on probing combined with a PD ≥ 5 mm and bone loss ≥2 mm. 

### 3.4. Smokers Definitions

The threshold for smoker definition varies: Alqahtani B and Javed define smokers as individuals smoking at least 1 cigarette daily for at least 1 year. Alqahtani A defines a moderate smoker as an individual smoking at least 11 cigarettes daily. Al Deeb defines smokers as an individual smoking for the last two years Fernandes-Costa do not describe the smoking status definition. Machtei does not describe the smoking status definition. C.M. de Waal does not describe the smoking status definition.

### 3.5. Risk of Bias in Studies 

The following domains were assessed: selection (1); comparability (2); and outcome (3). A total of one study resulted as having a high risk of bias (Javed), a total of five studies resulted as having a medium to high risk of bias. In all these studies the selection domain was the most concerning as none of them explicitly reports how the selection process was carried out.

A total of one study resulted as having a medium risk of bias (Alqahtani A) (Table 3).

### 3.6. Effects of Interventions

The results of individual studies are available in Table 4 and in the Appendix A.

### 3.7. Treatment of Peri-Implant Mucositis

Mechanical debridement: (Alqahtani B, Javed).

The use of mechanical debridement (MD) in the treatment of PiM causes an improvement in PD and BoP in nonsmokers.

In smokers there was no statistically significant difference in all parameters at all-time intervals.

Mechanical debridement + probiotic therapy: (Alqahtani B).

The use of mechanical debridement combined with probiotic therapy causes an improvement in Pi, BoP and PD in nonsmokers.

In smokers there was no statistically significant difference in all parameters at all-time intervals.

MD + adjunctive photodynamic therapy: (Javed).

The use of MD with adjunctive photodynamic therapy (aPTD) in the treatment of PiM causes an improvement in PD and BoP in nonsmokers.

In smokers there was no statistically significant difference in all parameters at all-time intervals except for a reduction of PD at six months.

Compared with MD alone, MD + aPTD shows better BoP at six months, but similar values at 12 months.

There was no statistically significant difference in CBL and PD among nonsmokers treated with MD and MD + aPTD.

Mechanical debridement + hygiene: (Fernandes-Costa).

Smoking did not present a significant association with “worsening” or “improvement” of clinical parameters. The treatment was not sufficient for the resolution of the condition.

### 3.8. Treatment of Peri-Implantitis

Mechanical debridement: (Alqahtani A).

In never-smokers PI, BoP and PD were significantly high at baseline compared with six-months’ follow-up. 

In never smokers there was no significant difference in peri-implant CBL.

In smokers there was no statistically significant difference in all parameters at all-time intervals.

Mechanical debridement + adjunctive photodynamic therapy: (Alqahtani A + Al Deeb).

Alqahtani found no statistically significant difference in all parameters at all-time intervals in smokers, while Al Deeb reports a significant reduction in PI, PD and a reduction of BoP in smokers at six months follow-up. BoP reduction was more evident in nonsmokers.

Mechanical debridement + chlorexidine chips: (Machtei).

Non-smoking patients showed significantly greater mean PD reductions at week 24 as compared with nonsmokers.

MD + biweekly debridement: (Machtei).

Nonsmokers showed higher values of all parameters at baseline as compared with follow-up time.

Smokers showed smaller reduction of all parameters at follow-up time as compared with baseline.

MD + resective surgery and chemical decontamination: (C.M. de Waal).

Smoking is a strong negative prognostic indicator for the outcome of resective surgical peri-implantitis treatment. 

The percentage of success with a bone loss <3 mm was approximately 80% in nonsmokers and 40% in smokers.

The percentage of success with a bone loss between 3 and 5 mm was approximately 70% in nonsmokers and 50% in smokers.

The percentage of success with a bone loss >5 mm was approximately 50% in nonsmokers and less than 20% in smokers.

## 4. Discussion

MD is a traditional protocol for the treatment of periodontal and peri-implant disease [18], however several adjunct therapies have been proposed and studied to enhance the healing of periodontal and peri-implant tissues.

Smoking is a common habit that deeply alters biological responses, and it is associated with an increased risk for periodontal and peri-implant disease as tissues are strongly interested by some substances contained in cigarettes, especially by nicotine, because of their weak and terminal vascularization. 

It is also hypothesized that the smoking habit may be related with a worse oral hygiene and a lack of attention in maintenance after treatment [13,26,27,28,29,30,31,32].

It is then crucial to understand which treatment is the most suitable for the population of smokers affected by peri-implant disease and if any of the proposed adjunctive therapies significantly improves clinical parameters.

The present review is the first to assess the influence of smoking on peri-implant clinical parameters after various kind of treatment.

### 4.1. Peri-Implant Mucositis

From the analysis of the included studies it appears that smoking might make patients affected by peri-implant mucositis refractory to the following therapies examined: mechanical debridement alone and mechanical debridement with adjunct probiotic therapy, while the same treatment in a sample of never smokers caused an improvement in all parameters. 

From the study published by Javed it appears that mechanical debridement with adjunct photodynamic therapy (MD + aPTD) causes a reduction of probing depth at six months, all the other parameters showed no significant differences.

Fernandes-Costa found no significant correlation between smoking and a worsening or improvement of clinical parameters, however they reported that they did not obtain complete resolution of the condition in any of the groups at longest follow-up time (54 months) with only mechanical debridement and supportive therapy, but a reduction in all clinical parameters was observed. 

A complete analysis was not feasible because in the study the number of smoking patients is not reported and all the data are reported for number of implants.

Obtaining a perfect hygiene is crucial in the prognosis of peri-implant mucositis [20].

Unfortunately, some of the studies do not consider plaque index and do not report if oral hygiene conditions differed from smokers and nonsmokers in the examined studies during the follow-up time. Also, none of the study reports if smokers kept the habit or reduced the number of cigarettes per day.

Alqahtani B suggests that regardless of how efficiently dental therapy is performed, routine postoperative oral hygiene maintenance plays a fundamental role in the long-term survival of dental treatment and oral health maintenance. 

### 4.2. Peri-Implantitis

From the analysis of the included studies it appears that smoking might make patients affected by peri-implantitis refractory to the following therapies examined: mechanical debridement alone, mechanical debridement with adjunct CHX chips, mechanical debridement with adjunct biweekly debridement and mechanical debridement with resective surgery and chemical decontamination.

From the study published by Al Deeb it appears that mechanical debridement with adjunctive photodynamic therapy causes a significant reduction in plaque index, probing depth and bleeding on probing in the group of smokers at six months follow-up. Bleeding on probing reduction was more evident in nonsmokers and e-cigarette smokers.

Bleeding on probing needs to be considered carefully, in many studies smokers had a lower percentage of bleeding sites at T0 as compared to nonsmokers, but this might be due to the fact that, like in periodontally compromised teeth, smoking ‘masks’ some inflammation signs because, as reported above, it reduces the blood supply in the periodontal and peri-implant tissues [32].

### 4.3. Applicability of Evidence

This review presents significant limitations such as the small number of studies that qualified as eligible, the heterogeneity of parameters examined, the variety of treatment proposed and the concerning risk of bias.

The main concern with the evidence available is that most of the included studies aimed to compare different treatment in a sample of patients representative of the general population and the study was not specifically design to compare a group of smokers and a group of nonsmokers. 

This led to incomplete data and unclear reporting of variations between the two subgroups.

Furthermore different threshold were used for the definition of pathological conditions and the status of smoker.

The only adjunctive treatment that appears to improve clinical parameters both in smokers and nonsmokers is adjunctive photodynamic therapy, according to the results from the studies of Javed (peri-implant mucositis) and Al Deeb (peri-implantitis).

Javed considered a sample of 40 nonsmokers and 41 smokers while Al Deeb considered 25 nonsmokers and 25 cigarette/e-cig smokers.

In the results published by Al Deeb the group of e-cigarettes smokers and cigarette smokers only differed for a different reduction of BoP at follow-up time. The frequency of e-cigarettes smoking and the composition of the liquids used was not reported hence it is not possible to assess any different response between the two groups.

There is no sufficient evidence to determine different clinical responses between cigarette smokers, e-cigarette smokers and waterpipe users.

In the study published by Alqahtani A the variations at follow-up time of cigarettes smokers and waterpipe users were comparable.

Evidence is limited because of the small sample considered, the study design and the high risk of bias of the cited studies hence it is not possible to assess that adjunctive photodynamic therapy is the best treatment options for peri-implant disease in smokers, however the data reported are encouraging and further study should be carried out to evaluate the benefits of this treatment and the option of multiple interventions. 

Possibly quitting smoking might improve clinical parameters at follow-up, but the time of interruption needed to appreciate the possible modifications needs to be investigated.

In the included studies it is not always clear if patients were encouraged to stop smoking.

There is also no sufficient evidence to determine a correlation between the number of cigarettes smoked and the risk reported, and about the quantity smoked per cigarette.

Repeating treatment at different intervals and considering a longer follow-up time might also influence the outcome of therapy as some of the studies [50] showed a worsening of some parameters from the follow-up at 3 months (not considered in this review) and the successive follow-up. 

A good oral hygiene and the reduction of external risk factors are crucial in the prevention of peri-implant disease and prior to therapy.

## 5. Conclusions

The majority of studies selected reported no statistically significant difference in all parameters recorded at all time intervals in smokers treated for peri-implant disease, while positive results were obtained in nonsmokers using the same treatment.In two studies mechanical debridment with adjunctive photodynamic therapy was reported to be effective in improving clinical parameters in smokers as compared to mechanical debridment only.

The current findings suggest that smoking might affect the outcome of peri-implant disease treatment.

## Figures and Tables

**Table 1:** Databases and search terms.

Pubmed (no filters applied)	(“peri-implantiti *”[MeSH] OR “Dental Prosthesis, Implant-Supported”[Mesh] OR “Dental Implant *”[Mesh] OR peri implantitis [tiab] OR peri-implant mucositis [tiab] OR perimucositis [tiab] OR peri-mucositis [tiab] OR fixed prosthetic treatment [tiab] OR implant treatment failure [tiab]) AND (“Dental Scaling”[Mesh] OR “Therapeutics” [Mesh] OR “Periodontal Debridement”[Mesh] OR “Subgingival Curettage”[Mesh] OR “Decontamination”[Mesh] OR “Anti-Bacterial Agents”[Mesh] OR scaling [tiab] OR curettage [tiab] OR debridement [tiab] OR root planing [tiab] OR laser decontamination [tiab] OR open flap debridement [tiab] OR surface decontamination [tiab] OR therapy [tiab] OR surgery [tiab] or treatment [tiab]) AND (“Smoking”[Mesh] OR smok * [tiab] OR cigarett * [tiab] OR tobacco [tiab])
Cochrane library (All text)	#1: (peri-implantitis OR peri-implantitis OR peri implantitis OR peri-implant mucositis OR peri implant mucositis OR perimucositis OR peri-mucositis OR dental implant) #2: (therapeutics OR scaling OR curettage OR debridement OR root planing OR decontamination OR laser decontamination OR open flap debridement OR surface decontamination OR therapy OR surgery OR treatment) #3: (Smoking OR smoke OR smoker OR cigarette OR cigarettes OR tobacco)
Scopus (all fields, no filters applied)	(peri-implantitis OR peri-implantitis OR peri-implant mucositis OR peri implantitis OR perimucositis OR peri implant mucositis OR peri-mucositis OR dental implant) AND (therapeutics OR scaling OR curettage OR debridement OR root planing OR decontamination OR laser decontamination OR open flap debridement OR surface decontamination OR therapy OR surgery OR treatment) AND (Smoking OR smoke OR smoker OR cigarette OR cigarettes OR tobacco)
Clinicaltrials.gov	condition/disease: peri-implantitis other terms: smoke OR tobacco Study type: clinical trials Study results: all studies Eligibility criteria: adults and older adults Sex: all

“*” indicated a specific syntax for queries.

**Table 2:** Description of included studies. Q = journal impact factor quartile, study arms: number of treatments considered in the study.

First Author	Nationality	Journal	Q	Study Arms	Treatment	Nonsmokers	Cigarette Smokers	e-Smoke/Other
Alqahtani 2019 [50]	Saudi Arabia and USA	Photodiagnosis and photodynamic therapy	Q3	2	Mechanical debridement (MD) with adjunct antimicrobial photodynamic therapy (aPDT)	16	17	32 waterpipe users
					Mechanical debridement (MD)	16	17	32 waterpipe users
Alqahtani 2019 [51]	Saudi Arabia, Pakistan and USA	Clinical Implant Dentistry and Related Research	Q1	2	Mechanical debridement (MD) with adjunctive probiotic therapy (PT) (ALL PIM)	20	20	0
					Mechanical debridement (MD) (ALL PIM)	20	20	0
Javed 2016 [52]	Saudi Arabia and USA	Photodiagnosis and photodynamic therapy	Q3	2	Mechanical debridement (MD) with adjunct antimicrobial photodynamic therapy (aPDT) (ALL PIM)	40	41	0
					Mechanical debridement (MD) (ALL PIM)	42	43	0
Al Deeb 2020 [53]	Saudi Arabia	Photodiagnosis and photodynamic therapy	Q3	1	Full-mouth mechanical debridement with adjunctive methylene blue-mediated PDT	25	25 cs + 25 e-smokers /vapers	25
Fernandes-Costa 2019 [54]	Brazil	Clinical Implant Dentistry and Related Research	Q1	1	Peri-implant supportive therapy (ALL PIM)	103 (implants)	28 (implants)	0
Machtei 2020 [55]	Europe, North America, Asia	Journal of periodontology	Q1	2	subgingival debridement (baseline and 12 weeks) + biweekly implant surface debridement and chlorexidine gluconate 2.5 mg chip (CHX chips) for 12 weeks	118	13	0
					subgingival debridement (baseline and 12 weeks) + biweekly implant surface debridement for 12 weeks	121	11	0
C.M de Waal 2015 [56]	Netherlands	Clinical Oral implants research	Q1	1	resective surgery + mechanical debridement and chemical decontamination of the implant surface	53	24	0

**Table 3:** Risk of bias in studies.

First Author	Domains									Results
	1-Selection				2-Comparability		3-Outcome			Total
	a	b	c	d	a	a.1	a	b	c	Max 9
Alqahtani 2019 [50]	0	1	1	1	1	0	1	1	1	7
Alqahtani 2019 [51]	0	0	0	1	1	1	1	1	1	6
Javed 2016 [52]	0	0	0	1	1	0	1	1	1	5
Al Deeb 2020 [53]	0	0	0	1	1	1	1	1	1	6
Fernandes-Costa 2019 [54]	0	0	0	1	1	1	1	1	1	6
Machtei 2020 [55]	0	0	0	1	1	1	1	1	1	6
C.M de Waal 2015 [56]	0	0	0	1	1	1	1	1	1	6

**Table 4:** Outcome measures of each individual study.

First Author						Alqahtani [50]		Alqahtani [51]		Javed [52]		Al Deeb [53]	Fernandes-Costa [54]	Machtei [55]		C.M de Waal [56]
Year						2019		2019		2016		2020	2019	2020		2015
Nationality						Saudi Arabia, Pakistan and USA		Saudi Arabia and USA		Saudi Arabia and USA		Saudi Arabia	Brazil	Europe, North America, Asia		Netherlands
Journal						Clinical Implant Dentistry and Related Research		Photodiagnosis and photodynamic therapy		Photodiagnosis and photodynamic therapy		Photodiagnosis and photodynamic therapy	Clinical Implant Dentistry and Related Research	Journal of periodontology		Clinical Oral implants research
Q						Q1		Q3		Q3		Q3	Q1	Q1		Q1
Study arms						2		2		2		1	1	2		1
treatment						Mechanical debridement (MD) with adjunctive probiotic therapy (PT) (ALL PIM)	Mechanical debridement (MD) (ALL PIM)	Mechanical debridement (MD) with adjunct antimicrobial photodynamic therapy (aPDT)	Mechanical debridement (MD)	Mechanical debridement (MD) with adjunct antimicrobial photodynamic therapy (aPDT) (ALL PIM)	Mechanical debridement (MD) (ALL PIM)	Full-mouth mechanical debridement with adjunctive methylene blue-mediated PDT	Peri-implant supportive therapy (ALL PIM)	subgingival debridment (baseline and 12 weeks) + biweekly implant surface debridment and chlorexidine gluconate 2.5 mg chip (CHX chips) for 12 weeks	subgingival debridment (baseline and 12 weeks) + biweekly implant surface debridment for 12 weeks	resective surgery + mechanical debridment and chemical decontaminationof the implant surface
Non smokers						20	20	16	16	40	42	25	103 (implants)	118	121	53
Cigarette smokers						20	20	17	17	41	43	25 cs + 25 e-smokers /vapers	28 (implants)	13	11	24
e-smoke/other						0	0	32 waterpipe users	32 waterpipe users	0	0	25	0	0	0	0
Reference standard			PD			yes	yes	yes	yes	yes	yes	yes	yes	yes		yes
			PI			yes	yes	yes	yes	no	no	yes	yes	no		no
			gingival bleeding index			no	no	no	no	no	no	no	yes	no		no
			BoP			yes	yes	yes	yes	yes	yes	yes	yes	yes		yes
			peri-implant sulcular fluid			no	no	no	no	no	no	yes	no	no		no
			Radiographic CBL			yes	yes	yes	yes	yes	yes	no	no	no		yes
			all variables			PD, PI and BoP Radiographical CBL	PD, PI and BoP Radiographical CBL	PI, BoP, PD and CBL	PI, BoP, PD and CBL	BoP, PD, CBL, Radiographical evaluation	BoP, PD, CBL, Radiographical evaluation	PI, PD, BoP, Peri-implant sulcular fluid	VPI (visible plaque index), GBI (gingival bleeding index), PD and BoP	PD, BoP		Pd, BoP, CBL
Measured effect	T 0	smokers	PD			4.2 ± 0.3	4.2 ± 0.4	5.2 ± 0.4 mm	5.2 ± 0.4 mm	5.51	5.21	5.8 ± 1.7	x	x	x	x
			PI			54.6 ± 10.2	54.6 ± 10.3	54.6 ± 12.2%	54.6 ± 12.2%	X	X	48.8 ± 11.4	x	x	x	x
			gingival bleeding index			x	X	X	X	X	X	X	x	x	x	x
			BoP			18.5 ± 3.1	18.5 ± 3.2	12.7 ± 2.6%	12.7 ± 2.6%	11.46	11.21	31.7 ± 6.2	x	x	x	x
			Peri-implant sulcular fluid			X	X	X	X	X	X	813 ± 388 1485 ± 794	X	X	X	X
			Radiographic CBL			1.5 ± 0.1 M 1.7 ± 0.2 D	1.5 ± 0.1 M 1.7 ± 0.2 D	5.2 ± 0.3 mm	5.2 ± 0.3 mm	2.17	2.05	X	X	X	X	X
		non smokers	PD			3.5 ± 0.2	3.5 ± 0.3	4.5 ± 0.2 mm	4.5 ± 0.2 mm	5.13	4.87	5.4 ± 0.9	/	x	x	x
			PI			48.3 ± 6.2	48.3 ± 6.3	39.6 ± 6.7%	39.6 ± 6.7%	X	X	46.4 ± 12.3	x	x	x	x
			gingival bleeding index			X	X	X	X	X	X	X	x	x	x	x
			BoP			50.5 ± 7.3	50.5 ± 7.4	44.1 ± 6.3%	44.1 ± 6.3%	29.84	32.45	47.1 ± 16.3	x	x	x	x
			Peri-implant sulcular fluid			X	X	X	X	X	X	789 ± 361 1269 ± 648	x	x	x	x
			Radiographic CBL			1.2 ± 0.4 M 1.2 ± 0.3 D	1.2 ± 0.4 M 1.2 ± 0.3 D	4.3 ± 0.2 mm	4.3 ± 0.2 mm	2.15	1.97	X	x	x	x	x
Measured effect	24 weeks	smokers	PD			x	x	x	x	x	x	x	x	PD reduction:1.08 ± 1.26	PD reduction: 1.55 ± 1.21	x
			PI			x	x	x	x	X	X	x	x	x	x	x
			gingival bleeding index			x	x	x	x	X	X	x	x	x	x	x
			BoP			x	x	x	x	x	x	x	x	x	x	x
			Peri-implant sulcular fluid			x	x	x	x	X	X	x	x	x	x	x
			Radiographic CBL			x	x	x	x	x	x	x	x	x	x	x
		non smokers	PD			x	x	x	x	x	x	x	x	PD reduction: 1.86 ± 1.09	PD reduction: 1.51 ± 1.13	x
			PI			x	x	x	x	X	X	x	x	x	x	x
			gingival bleeding index			x	x	x	x	X	X	x	x	x	x	x
			BoP			x	x	x	x	x	x	x	x	x	x	x
			Peri-implant sulcular fluid			x	x	x	x	X	X	x	x	x	x	x
			Radiographic CBL			x	x	x	x	x	x	x	x	x	x	x
	six months	smokers	PD			3.6 ± 0.3	3.4 ± 0.5	4.6 ± 0.2 mm	4.4 ± 0.3 mm	2.49	4.09	3.7 ± 1.7	x	x	x	x
			PI			50.6 ± 12.2	50.4 ± 5.8	46.5 ± 7.3%	43.7 ± 8.2%	X	X	16.1 ± 2.2	x	x	x	x
			gingival bleeding index			X	X	X	X	X	X	X	x	x	x	x
			BoP			13.2 ± 2.5	14.4 ± 2.8	12.6 ± 3.8%	13.5 ± 5.6%	8.07	10.16	20.4 ± 4.8	x	x	x	x
			Peri-implant sulcular fluid			X	X	X	X	X	X	767 ± 562 1364 ± 1032	X	X	X	X
			Radiographic CBL			Not assessed	Not assessed	5 ± 0.2 mm	5.1 ± 0.3 mm	2.25	2.16	X	X	X	X	X
		non smokers	PD			2.6 ± 0.3	3.3 ± 0.5	2.4 ± 0.5 mm	2.6 ± 0.4 mm	2.35	3.94	3.0 ± 1.7	x	x	x	x
			PI			27.3 ± 5.1	30.2 ± 4.6	14.1 ± 3.1%	23.4 ± 3.5%	X	X	18.2 ± 4.3	x	x	x	x
			gingival bleeding index			X	X	X	X	X	X	X	x	x	x	x
			BoP			24.2 ± 3.4	33.2 ± 3.4	8.2 ± 1.5%	20.8 ± 4.1%	10.12	22.02	18.5 ± 5.3	x	x	x	x
			Peri-implant sulcular fluid			X	X	X	X	X	X	445 ± 324, 1173 ± 956	X	X	X	X
			Radiographic CBL			Not assessed	Not assessed	3.3 ± 0.4 mm	4.1 ± 0.2 mm	2.14	2.07	X	X	X	X	X
Measured effect	12 months	smokers	PD			x	x	x	x	2.47	3.18	x	x	x	x	success % per mm of initial bone loss: 1–3 = 43.28; 3–5 = 54.8; >5 = 20.8
			PI			x	x	x	x	X	X	x	x	x	x	x
			gingival bleeding index			x	x	x	x	X	X	x	x	x	x	x
			BoP			x	x	x	x	11.34	11.87	x	x	x	x	x
			Peri-implant sulcular fluid			x	x	x	x	X	X	x	x	x	x	x
			Radiographic CBL			x	x	x	x	2.36	2.26	x	x	x	x	x
		non smokers	PD			x	x	x	x	2.33	2.94	x	x	x	x	success % divided per mm of initial bone loss: 1–3 = 84; 3–5 = 65.5; >5 = 45.1
			PI			x	x	x	x	X	X	x	x	x	x	x
			gingival bleeding index			x	x	x	x	X	X	x	x	x	x	x
			BoP			x	x	x	x	14.98	14.20	x	x	x	x	x
			Peri-implant sulcular fluid			x	x	x	x	X	X	x	x	x	x	x
			Radiographic CBL			x	x	x	x	2.35	2.14	x	x	x	x	x
	54 months	smokers	PD			x	x	x	x	x	x	x	worsening% = 46.4 improvement % = 53.6	x	x	x
			PI			x	x	x	x	X	X	x	x	x	x	x
			gingival bleeding index			x	x	x	x	X	X	x	x	x	x	x
			BoP			x	x	x	x	x	x	x	worsening% = 46.4 improvement % = 53.6	x	x	x
			Peri-implant sulcular fluid			x	x	x	x	X	X	x	x	x	x	x
			Radiographic CBL			x	x	x	x	x	x	x	x	x	x	x
		non smokers	PD			x	x	x	x	x	x	x	worsening% = 54.4 improvement % = 45.6	x	x	x
			PI			x	x	x	x	X	X	x	x	x	x	x
			gingival bleeding index			x	x	x	x	X	X	x	x	x	x	x
			BoP			x	x	x	x	x	x	x	worsening%= 59.2 improvement % = 40.8	x	x	x
			Peri-implant sulcular fluid			x	x	x	x	X	X	x	x	x	x	x
			Radiographic CBL			x	x	x	x	x	x	x	x	x	x	x

## Data Availability

The data that support the findings of this study are available from the corresponding author upon reasonable request.

## Appendix A. Results of Individual Studies

Alqahtani (b): Treated peri-implant mucositis using Mechanical debridement (MD) with or without adjunctive probiotic therapy (PT).

Among cigarette-smokers there was no statistically significant difference in peri-implant PI, BoP, and PD at all-time intervals with and without adjunct PT.

In never smokers the total mean values of PI, BoP, and PD were significantly higher at baseline compared with their respective scores at six-months’ follow-up among individuals that underwent MD with and without adjunct PT.

At six-months’ follow- up, there was no statistically significant difference in mean PI, BoP, and PD among individuals that underwent MD with or without adjunct PT.

Alqahtani (a): Treated peri-implantitis using Mechanical debridement (MD) with or without adjunct antimicrobial photodynamic therapy (aPDT)

Among cigarette smokers and waterpipe users there was no statistically significant difference in PI, BoP, PD and CBL at six-months’ follow-up compared with baseline.

In never-smokers treated with MD only and MD+aPTD, PI, BoP and PD were significantly high at baseline compared with six-months’ follow-up.

There was no significant difference in peri-implant CBL among individuals that underwent MD with or without adjunct aPDT.

Results were comparable for cigarette smokers and waterpipe users.

Javed: Treated peri-implant mucositis using Mechanical debridement (MD) with (test) or without (control) adjunct antimicrobial photodynamic therapy (aPDT):

Among smokers there was no statistically significant difference in BoP in both treatment groups at 6-and 12-months of follow-up compared with baseline.

Among smokers PD was statistically significantly higher at baseline as compared with 6- and 12- months of follow-up in both test and control group.

In nonsmokers BoP and PD were significantly higher among individuals in the test and control groups at baseline compared with 6 and 12-months follow-up.

At six-months of follow-up BoP was statistically significantly higher among individuals in the test group compared with control group.

At 12-months follow-up, there was no statistically significant difference in BoP among patients in the test and control-groups.

There was no statistically significant difference in PD among nonsmokers in the test and control-groups at 12-months of follow-up

There was no statistically significant difference in CBL among smokers and nonsmokers in the test and control groups at all time intervals

Al Deeb: Treated peri-implantitis using full-mouth mechanical debridement with adjunctive methylene blue-mediated PDT in cigarette smokers, e-cig smokers and nonsmokers.

All three groups showed significant reduction in PI from baseline to six months.

All groups showed significant reduction of BoP at six months, with e-cig Group and nonsmokers showing further reduction in BoP (*p* < 0.05).

Mean PD significantly reduced in all the groups at six months, showing no statistically significant difference between the groups at any time-point.

Nonsmokers demonstrated statistically significantly reduced mean RANK-L levels at six months.

No significant change was observed for biological bone biomarkers among tobacco smokers.

Fernandes-Costa: Treated peri-implant mucositi using peri-implant supportive therapy (implant decontamination, scaling, and root planing with metallic and plastic curettes when necessary and the use of dental floss) associated with oral hygiene instructions (related to each type of prosthesis). The implementation of peri-implant support therapy was not sufficient for the resolution of peri-implant mucositis, although reductions in clinical parameters with respect to baseline were observed and maintained during follow-up.

Smoking did not present a significant association with “worsening” or “improvement” of clinical parameters.

Machtei: Treated peri-implantitis using subgingival debridment with or without chlorexidine gluconate 2.5 mg chip (CHX chips applied at baseline and 12 weeks).

A complete separate analysis for smokers and nonsmokers was not reported.

Non-smoking patients in the experimental groups showed significantly greater mean IPD reductions at week 24 as compared with the control nonsmokers.

Patients in the control group that were nonsmokers showed higher values of all parameters at baseline as compared with follow-up time.

C.M de Waal: Treated peri-implantitis using resective surgery with mechanical debridment and chemical decontamination of the implant surface.

The amount of bone loss at baseline and smoking are strong prognostic indicators for the outcome of resective surgical peri-implantitis treatment.

The percentage of success with a bone loss <3 mm was approximately 80% in nonsmokers and 40% in smokers.

The percentage of success with a bone loss between 3 and 5 mm was approximately 70% in nonsmokers and 50% in smokers.

The percentage of success with a bone loss >5 mm was approximately 50% in nonsmokers and less than 20% in smokers.

## References

[1] Buser D., Sennerby L., De Bruyn H. (2017). Modern implant dentistry based on osseointegration: 50 years of progress, current trends and open questions. Periodontol. 2000.

[2] Moraschini V., Poubel L.D.C., Ferreira V., Barboza E.D.S. (2015). Evaluation of survival and success rates of dental implants reported in longitudinal studies with a follow-up period of at least 10 years: A systematic review. Int. J. Oral Maxillofac. Surg..

[3] Vande A., Sanyal P., Nilesh K. (2022). Effectiveness of the photobiomodulation therapy using low-level laser around dental implants: A systematic review and meta-analysis. Dent. Med. Probl..

[4] Esposito M., Grusovin M.G., Worthington H. (2012). Interventions for replacing missing teeth: Treatment of peri-implantitis. Cochrane Database Syst. Rev..

[5] Krawiec M., Olchowy C., Kubasiewicz-Ross P., Hadzik J., Dominiak M. (2022). Role of implant loading time in the prevention of marginal bone loss after implant-supported restorations: A targeted review. Dent. Med. Probl..

[6] Grusovin M.G., Coulthard P., Worthington H.V., George P., Esposito M. (2010). Interventions for replacing missing teeth: Maintaining and recovering soft tissue health around dental implants. Cochrane Database Syst. Rev..

[7] Caton J.G., Armitage G., Berglundh T., Chapple I.L., Jepsen S., Kornman K.S., Mealey B.L., Papapanou P.N., Sanz M., Tonetti M.S. (2018). A new classification scheme for periodontal and peri-implant diseases and conditions—Introduction and key changes from the 1999 classification. J. Periodontol..

[8] Schwarz F., Derks J., Monje A., Wang H.-L. (2018). Peri-Implantitis. J. Clin. Periodontol..

[9] Tamrakar A.K., Murali G., Singh S., Shakila R., Shyam S. (2020). Evaluation of subgingival microbiota around single tooth implants. J. Oral Biol. Craniofacial Res..

[10] Kormas I., Pedercini C., Pedercini A., Raptopoulos M., Alassy H., Wolff L.F. (2020). Peri-Implant Diseases: Diagnosis, Clinical, Histological, Microbiological Characteristics and Treatment Strategies. A Narrative Review. Antibiotics.

[11] Galárraga-Vinueza M.E., Tangl S., Bianchini M., Magini R., Obreja K., Gruber R., Schwarz F. (2020). Histological characteristics of advanced peri-implantitis bone defects in humans. Int. J. Implant Dent..

[12] Hämmerle C.H.F., Tarnow D. (2018). The etiology of hard- and soft-tissue deficiencies at dental implants: A narrative review. J. Peri-odontol..

[13] Dreyer H., Grischke J., Tiede C., Eberhard J., Schweitzer A., Toikkanen S.E., Glöckner S., Krause G., Stiesch M. (2018). Epidemiology and risk factors of peri-implantitis: A systematic review. J. Periodontal Res..

[14] Berglundh T., Armitage G., Araujo M.G., Avila-Ortiz G., Blanco J., Camargo P.M., Chen S., Cochran D., Derks J., Figuero E. (2018). Peri-implant diseases and conditions: Consensus report of workgroup 4 of the 2017 World Workshop on the Classification of Periodontal and Peri-Implant Diseases and Conditions. J. Clin. Periodontol..

[15] Maurício J.M., Miranda T.S., Almeida M.L., Silva H.D., Figueiredo L.C., Duarte P.M. (2019). An umbrella review on the effects of diabetes on implant failure and peri-implant diseases. Braz. Oral Res..

[16] Rokaya D., Srimaneepong V., Wisitrasameewon W., Humagain M., Thunyakitpisal P. (2020). Peri-Implantitis Update: Risk Indi-cators, Diag-Nosis, and Treatment. Eur. J. Dent..

[17] Poli P.P., Beretta M., Grossi G.B., Maiorana C. (2016). Risk indicators related to peri-implant disease: An observational retrospective cohort study. J. Periodontal Implant Sci..

[18] Del Amo F.S., Yu S.-H., Wang H.-L. (2016). Non-Surgical Therapy for Peri-Implant Diseases: A Systematic Review. J. Oral Maxillofac. Res..

[19] Bianco L.L., Montevecchi M., Ostanello M., Checchi V. (2021). Recognition and treatment of peri-implant mucositis: Do we have the right perception? A structured review. Dent. Med. Probl..

[20] Khan A., Goyal A., Currell S.D., Sharma D. (2020). Management of Peri-Implantitis Lesions without the Use of Systemic Antibiotics: A Systematic Review. Dent. J..

[21] Mahato N., Wu X., Wang L. (2016). Management of peri-implantitis: A systematic review, 2010–2015. SpringerPlus.

[22] Khoury F., Keeve P.L., Ramanauskaite A., Schwarz F., Koo K.-T., Sculean A., Romanos G. (2019). Surgical treatment of peri-implantitis—Consensus report of working group 4. Int. Dent. J..

[23] Renvert S., Quirynen M. (2015). Risk indicators for peri-implantitis. A narrative review. Clin. Oral Implant. Res..

[24] Courtney R. (2015). The Health Consequences of Smoking-50 Years of Progress: A Report of the Surgeon General, 2014Us Department of Health and Human Services Atlanta, GA: Department of Health and Human Services, Centers for Disease Control and Prevention, National Center for: Critique. Drug Alcohol Rev..

[25] Pagano S., Negri P., Coniglio M., Bruscoli S., Di Michele A., Marchetti M.C., Valenti C., Gambelunghe A., Fanasca L., Billi M. (2021). Heat-not-burn tobacco (IQOS), oral fibroblasts and keratinocytes: Cytotoxicity, morphological analysis, apoptosis and cellular cycle. An in vitro study. J. Periodontal Res..

[26] Jansson L., Lavstedt S. (2002). Influence of Smoking on Marginal Bone Loss and Tooth Loss--a Prospective Study over 20 Years: Effects of Smoking on Bone/Tooth Loss. J. Clin. Periodontol..

[27] Chen H., Liu N., Xu X., Qu X., Lu E. (2013). Smoking, Radiotherapy, Diabetes and Osteoporosis as Risk Factors for Dental Implant Failure: A Meta-Analysis. PLoS ONE.

[28] Chrcanovic B.R., Albrektsson T., Wennerberg A. (2015). Smoking and dental implants: A systematic review and meta-analysis. J. Dent..

[29] Moraschini V., Barboza E.D.P. (2016). Success of dental implants in smokers and non-smokers: A systematic review and meta-analysis. Int. J. Oral Maxillofac. Surg..

[30] Windael S., Vervaeke S., De Buyser S., De Bruyn H., Collaert B. (2020). The Long-Term Effect of Smoking on 10 Years’ Survival and Success of Dental Implants: A Prospective Analysis of 453 Implants in a Non-University Setting. J. Clin. Med..

[31] Qian J., Wennerberg A., Albrektsson T. (2012). Reasons for Marginal Bone Loss around Oral Implants. Clin. Implant Dent. Relat. Res..

[32] Johnson G.K., Guthmiller J.M. (2007). The impact of cigarette smoking on periodontal disease and treatment. Periodontol. 2000.

[33] Akram Z., Vohra F., Bukhari I.A., Sheikh S.A., Javed F. (2018). Clinical and Radiographic Peri-Implant Parameters and Proin-flammatory Cyto-Kine Levels among Cigarette Smokers, Smokeless Tobacco Users, and Nontobacco Users. Clin. Implant Dent. Relat. Res..

[34] Abdellatif H., Binshabaib M., Shawky H., Alharthi S. (2021). Association between Periodontitis and Genetic Polymorphisms in Interleukins among Patients with Diabetes Mellitus. Dent. J..

[35] Abduljabbar T. (2017). Effect of Mechanical Debridement with Adjunct Antimicrobial Photodynamic Therapy in the Treatment of Pe-Ri-Implant Diseases in Type-2 Diabetic Smokers and Non-Smokers. Photodiagnosis Photodyn. Ther..

[36] Keenan J.R., Veitz-Keenan A. (2016). The Impact of Smoking on Failure Rates, Postoperative Infection and Marginal Bone Loss of Dental Implants: Question: In Patients Undergoing Implant Placement, Are Patients Who Smoke Compared with Those Who Do Not at Higher Risk for Implant Failure, Postoperative Infection and Greater Marginal Bone Loss?. Evid.-Based Dent..

[37] Gazibara T., Milic M., Parlic M., Stevanovic J., Mitic N., Maric G., Tepavcevic D.K., Pekmezovic T. (2021). What differs former, light and heavy smokers? Evidence from a post-conflict setting. Afr. Health Sci..

[38] Vatsa R., Kumar A., Nasreen S., Bandgar S., Bhowmick D., Priyadarshni P. (2021). Comparative evaluation of marginal bone loss and implant failure rate in smokers and nonsmokers. J. Pharm. Bioallied Sci..

[39] Binshabaib M., Alharthi S.S., Akram Z., Khan J., Rahman I., Romanos G.E., Javed F. (2019). Clinical periodontal status and gingival crevicular fluid cytokine profile among cigarette-smokers, electronic-cigarette users and never-smokers. Arch. Oral Biol..

[40] Kanmaz M., Kanmaz B., Buduneli N. (2021). Periodontal treatment outcomes in smokers: A narrative review. Tob. Induc. Dis..

[41] Pesce P., Menini M., Ugo G., Bagnasco F., Dioguardi M., Troiano G. (2022). Evaluation of periodontal indices among non-smokers, tobacco, and e-cigarette smokers: A systematic review and network meta-analysis. Clin. Oral Investig..

[42] Alharthi S.S., BinShabaib M., Akram Z., Rahman I., Romanos G.E., Javed F. (2019). Impact of cigarette smoking and vaping on the outcome of full-mouth ultrasonic scaling among patients with gingival inflammation: A prospective study. Clin. Oral Investig..

[43] Silva H. (2021). Tobacco Use and Periodontal Disease—The Role of Microvascular Dysfunction. Biology.

[44] Tatsumi M., Yanagita M., Yamashita M., Hasegawa S., Ikegami K., Kitamura M., Murakami S. (2021). Long-term exposure to cigarette smoke influences characteristics in human gingival fibroblasts. J. Periodontal Res..

[45] Chaffee B.W., Couch E.T., Vora M.V., Holliday R.S. (2021). Oral and periodontal implications of tobacco and nicotine products. Periodontology 2000.

[46] D’Ambrosio F., Pisano M., Amato A., Iandolo A., Caggiano M., Martina S. (2022). Periodontal and Peri-Implant Health Status in Traditional vs. Heat-Not-Burn Tobacco and Electronic Cigarettes Smokers: A Systematic Review. Dent. J..

[47] Caggiano M., Gasparro R., D’Ambrosio F., Pisano M., Di Palo M.P., Contaldo M. (2022). Smoking Cessation on Periodontal and Peri-Implant Health Status: A Systematic Review. Dent. J..

[48] Page M.J., McKenzie J.E., Bossuyt P.M., Boutron I., Hoffmann T.C., Mulrow C.D., Shamseer L., Tetzlaff J.M., Akl E.A., Brennan S.E. (2021). The PRISMA 2020 statement: An updated guideline for reporting systematic reviews. BMJ.

[49] Wells G., Shea B., O’connell J., Robertson J., Peterson J., Welch V. The Newcastle-Ottawa Scale (Nos) for Assessing the Quality of Non Randomised Studies in Meta-Analysis.

[50] Alqahtani F., Alqhtani N., Alkhtani F., Divakar D.D., Al-Kheraif A.A., Javed F. (2019). Efficacy of mechanical debridement with and without adjunct antimicrobial photodynamic therapy in the treatment of peri-implantitis among moderate cigarette-smokers and waterpipe-users. Photodiagnosis Photodyn Ther..

[51] Alqahtani F., Alqahtani M., Shafqat S.S., Akram Z., Al-Kheraif A.A., Javed F. (2019). Efficacy of mechanical debridement with adjunctive probiotic therapy in the treatment of peri-implant mucositis in cigarette-smokers and never-smokers. Clin. Implant Dent Relat Res..

[52] Javed F., Abduljabbar T., Carranza G., Gholamiazizi E., Mazgaj D.K., Kellesarian S.V., Vohra F. (2016). Efficacy of periimplant mechanical debridement with and without adjunct antimicrobial photodynamic therapy in the treatment of periimplant diseases among cigarette smokers and non-smokers. Photodiagnosis Photodyn Ther..

[53] Al Deeb M., Alresayes S., A Mokeem S., Alhenaki A.M., AlHelal A., Vohra F., Abduljabbar T. (2020). Clinical peri-implant health and biological bone marker levels in tobacco users treated with photodynamic therapy. Photodiagnosis Photodyn Ther..

[54] Fernandes-Costa A.N., Menezes K.M., Borges S.B., Roncalli A.G., Calderon P.D.S., de V Gurgel B.C. (2019). A prospective study of the clinical outcomes of peri-implant tissues in patients treated for peri-implant mucositis and followed up for 54 months. Clin. Implant Dent Relat Res..

[55] Machtei E.E., Romanos G., Kang P., Travan S., Schmidt S., Papathanasiou E., Tatarakis N., Tandlich M., Liberman L.H., Horwitz J. (2021). Repeated delivery of chlorhexidine chips for the treatment of peri-implantitis: A multicenter, randomized, comparative clinical trial. J. Periodontol..

[56] de Waal Y.C., Raghoebar G.M., Meijer H.J., Winkel E.G., van Winkelhoff A.J. (2016). Prognostic indicators for surgical peri-implantitis treatment. Clin. Oral Implants Res..

